# The voice of non-pregnant women on alcohol consumption during pregnancy: a focus group study among women in Sweden

**DOI:** 10.1186/s12889-015-2519-2

**Published:** 2015-11-30

**Authors:** Janna Skagerström, Elisabet Häggström-Nordin, Siw Alehagen

**Affiliations:** Department of Medical and Health Sciences, Division of Community Medicine, Linköping University, SE-581 83 Linköping, Sweden; Department of Women’s and Children’s Health, Uppsala University, SE-751 85 Uppsala, Sweden; School of Health, Care and Social Welfare, Mälardalen University, SE-721 23 Västerås, Sweden; Department of Medical and Health Sciences, Division of Nursing, Linköping University, SE-581 85 Linköping, Sweden

**Keywords:** Alcohol consumption, Pregnancy, Fertile age, Pregnancy planning, Health education, Focus group

## Abstract

**Background:**

Consensus is that fetal exposure to alcohol is harmful. Abstinence while trying to conceive and throughout pregnancy is recommended. Despite this, there are many women who consume alcohol around conception and until pregnancy recognition. The aim of this study was to explore the voice of non-pregnant women concerning alcohol consumption and its relation to pregnancy.

**Methods:**

Data were collected through seven focus groups interviews with 34 women of fertile age, who were neither pregnant nor mothers. Semi-structured interviews were undertaken, recorded and transcribed verbatim and then analysed using thematic analysis.

**Results:**

Three main themes were identified in the analysis: an issue that cannot be ignored; awareness and uncertainty concerning alcohol and pregnancy; and transition to parenthood. Alcohol was an integral part of the women’s lives. A societal expectation to drink alcohol was prevalent and the women used different strategies to handle this expectation. Most women agreed not to drink alcohol during pregnancy although their knowledge on the specific consequences was scanty and they expressed a need for more information. Most of the participants found drinking alcohol during pregnancy to be irresponsible and saw pregnancy as a start of a new way of life.

**Conclusions:**

Social expectations concerning women’s alcohol use change with pregnancy when women are suddenly expected to abstain. Although most study participants shared an opinion for zero tolerance during pregnancy, their knowledge regarding consequences of drinking during pregnancy were sparse. In order for prospective mothers to make informed choices, there is a need for public health initiatives providing information on the relationship between alcohol consumption and reproduction.

## Background

It is well known that alcohol consumption during pregnancy has negative effects on the developing fetus, as well as life-long effects on the child. Although there is consensus regarding the effects of a high level of alcohol consumption, research on the effects of exposure to small to moderate amounts show differing results [1]. The first period of pregnancy is a critical period for alcohol teratogenicity. The human brain is susceptible to insult from maternal alcohol intake as early as the third week of gestation [2]. Even small to moderate amounts of alcohol consumed in the first period of pregnancy have been found to increase the risk of spontaneous abortion [3].

Abstinence while trying to conceive has been recommended by several experts [4, 5] and to refrain or decrease alcohol intake during this critical period is the official policy in many European countries [6]. However, studies on alcohol consumption in the periconceptional period, i.e. the time around conception including early pregnancy, have shown that many women continue to drink alcohol until pregnancy recognition [4, 7, 8]. Sixty-eight percent of pregnant women in Sweden did not decrease their alcohol consumption until pregnancy recognition [9]. Reasons stated for an intention to keep drinking alcohol until pregnancy recognition included that consuming small amounts of alcohol was harmless and wanting to “keep having fun” [10]. Social norms regarding alcohol consumption might even influence pregnant women to drink during social occasions to avoid revealing that they are pregnant [11].

Research on views and knowledge regarding drinking during pregnancy has focused on women who are pregnant. For example, pregnant women who were knowledgeable about the risks of alcohol consumption drank to a lesser extent while pregnant [11–13]. Women meeting social norms advocating that drinking small amounts during pregnancy is a low risk activity were more likely to agree with those norms [10, 14].

In order to influence alcohol consumption around conception, there is a need to understand how non-pregnant women reason about alcohol in relation to pregnancy. However, we have not been able to identify any studies in a European setting where the views of non-pregnant women on alcohol use in relation to pregnancy have been investigated. Therefore, the aim of this study was to explore the voice of non-pregnant women concerning alcohol consumption in relation to pregnancy. This knowledge is potentially useful for the development of preventive interventions to achieve reduced alcohol exposure in early pregnancy.

## Methods

### Design

Focus group interviews are useful for identifying attitudes and social norms regarding a specific subject. The method allows interaction and learning between participants and offers the potential to gather information on group consensus or diversity [15, 16].

### Study setting and participants

In Sweden, the total alcohol consumption per woman over 15 years of age is about 6 litres of pure alcohol per year [17]. Almost all pregnant women in Sweden visit antenatal care where they are screened for alcohol consumption before pregnancy, given information on alcohol and pregnancy, and are recommended to abstain throughout pregnancy.

For this study, snowball recruitment and a convenient sample [18] were used. To enhance variety among participants, the recruitment took place at three different sites/locations in the south east of Sweden, one city with a university and two towns without university. Inclusion criteria were age 15–35 years, not pregnant, no children, and Swedish speaking.

For recruitment within school classes, information was sent to school principles. After their approval, teachers were contacted and the study was then presented verbally to pupils by their teacher or the first author of this study. Interested pupils left their contact information. Enough pupils were interested so that a focus group could be conducted in each class. Participants recruited at other locations were asked to provide the names of women in their network matching the inclusion criteria. These women were then approached until the groups were full. At recruitment, the women were given written information about the study. After recruitment, interested women were contacted via e-mail to schedule the interview.

The focus group interviews were conducted between September 2013 and February 2014, and held in easily accessible places, such as a library for the composite groups or a study room at school for the pre-existing groups.

### Data collection

A study-specific semi-structured interview guide was developed for the study. Areas covered in the interviews were perceptions regarding alcohol consumption in general and at different stages of pregnancy. Pictures were used as stimuli material to encourage interaction [19] and start discussions. The pictures showed an alcoholic drink, two women drinking alcohol, a positive pregnancy test in combination with an invitation to an office party, and a pregnant women reaching for a glass of wine. After introducing each picture, the moderator asked about the participant’s first thoughts and followed up with questions like “what do you think about the norms of alcohol consumption?” Later, a picture sorting exercise was applied [15]. The participants sorted magazine pictures of women into two categories, high and low risk of using alcohol during pregnancy, while thinking out loud.

In order to acquaint the moderator with the role and test the interview guide, two pilot focus group interviews were performed. Afterwards, minor revisions were made in the interview guide. The pilot interviews were not analysed for this study.

During all interviews, the first author acted as moderator, assisted by an assessor (PhD student with previous experience from qualitative interviewing). The moderator’s role was to explain the aim of the study, lead the interviews by introducing stimuli materials, ask questions and give all participants a chance to talk. The assessor took care of practical issues and took field notes [15]. The participants filled out an anonymous questionnaire with demographic data. In order to make the women feel relaxed, some small talk took place at the start of the interviews. The interviews were digitally recorded and lasted between 45 and 75 min (mean 61 min).

### Data analysis

The material was analysed using inductive thematic analysis according to Braun and Clarke [20] and included six steps. (1) The first author familiarized herself with the data by conducting and transcribing the interviews verbatim. The complete transcript was read by all authors. Interesting subjects in the material were written down and discussed. (2) An initial code was given to interesting features of the data that related to the study aim. The interviews were given equal attention and were coded for as many patterns as possible. (3) The codes were organized inductively into initial themes by the first author and subsequently revised jointly by all three authors. (4) The initial themes were reviewed in relation to the entire data. Coded extracts were moved to create coherent and consistent themes. Thematic maps were created and refined. (5) The themes were defined by identifying the core and writing down the content of each theme. All authors were involved in refining the definitions and finally labelling the themes. (6) When producing the article, the themes were described in the text and citations were chosen to support the points of the analysis.

### Ethical considerations

This study was approved by the Regional Ethical Review Board in Linköping (Dnr 2013/93–31). Before the interviews, the participants provided written consent to participate in the study and verbal consent to the interviews being recorded. The participants were aware that they could end their participation at any time. At the beginning and end of the interviews, they had the chance to ask the moderator questions. It was made clear that they would be de-identified in the written report. A website address with information on the effects of alcohol consumption during pregnancy was provided.

## Results

In total, 34 women participated in seven focus group interviews. Each group consisted of 3–8 participants, aged from 17 to 34 years (mean 23.6 years). For further information on the participants, see Table 1. Three interviews were conducted within school classes, two with high school students (aged 17–19 years), and one with students in adult education (aged 20–30 years). Four groups consisted of women recruited from other sites and via snowballing.

**Table 1:** Characteristics of the participants by group

	Group 1	Group 2	Group 3	Group 4	Group 5	Group 6	Group 7
No. of participants	3	3	5	8	5	5	5
Mean age, years (range)	24.3 (22–27)	23 (21–26)	18.4 (17–19)	17.9 (17–18)	23.4 (20–30)	30.4 (27–31)	31.2 (27–34)
Education							
Intermediate	0	0	5	8	5	0	3
University/college	3	3	0	0	0	5	2
Occupation							
Student	3	2	5	8	5	0	1
Employed	1	1	0	0	0	5	5
Sick leave	0	0	0	0	0	0	1
Marital status							
Married/cohabiting	1	1	1	0	4	1	3
In a relationship	0	2	1	2	0	3	3
Single	2	0	3	6	1	1	2

**Fig. 1:**
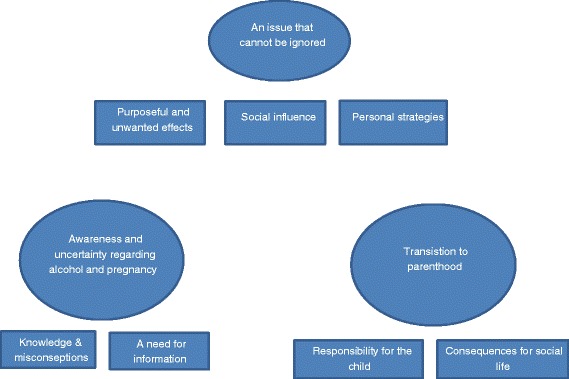
Thematic map illustrating themes and subthemes

Three themes and seven subthemes were identified in the thematic analyses of the data. The themes were an issue that cannot be ignored, awareness and uncertainty regarding alcohol and pregnancy, and transition to parenthood. The themes and subthemes are illustrated by a thematic map in Fig. 1. In the interviews, two of the participants stated that they never used alcohol, the rest drank differing quantities of alcohol at various frequencies.

### An issue that cannot be ignored

The participants described alcohol as an integral part of Swedish culture that they needed to relate to. They were aware of the potential harm of alcohol consumption, and some were critical about the prevalent drinking norm. However, most drank alcohol as it was considered standard practice in many social situations. Abstaining or drinking in a different manner than expected would not go unnoticed. Expectations on drinking were thought to depend on personal characteristics and stages in life. The women used a variety of strategies to handle expectations on alcohol consumption.

#### Purposeful and unwanted effects

Almost exclusively festive associations, like celebration and time of, were made to the stimuli picture of a drink. Alcohol can function as a way to connect people and create fellowship, for example among students or colleagues. Some of the women used alcohol to gain certain required characteristics such as becoming more social, relaxed or losing inhibitions. Drinking alcohol to relax was found acceptable by some women, whereas others saw this as a risky drinking behaviour.

*But I could just look at myself, I can drink to relax and to be a bit more impulsive and to take more risks because it’s nice and you can blame it on that (alcohol consumption) too. (participant 2; group 1)*

Binge drinking was often found to lead to unwanted consequences such as losing control, feeling nauseous, becoming aggressive or sad as well as having remorse the day after. Misuse or dependence was mentioned in all groups. Several women voiced that young people have a shared responsibility for an individual’s alcohol dependence by pushing each other to drink.

#### Social influence

Most of the women agreed that it was socially expected of them to drink. The predominant view was that if someone chose not to drink alcohol as expected, they would be asked about their reasons. If you choose not to drink, many will suspect that you are pregnant. Some women described direct pressure whereby non-drinkers were believed to be uninteresting and should be persuaded to join the drinking occasion. However, other women did not feel questioned when choosing not to drink. A trend towards drinking less frequently and/or smaller amounts was mentioned.

To drink in social situations, at a good dinner or to drink at the same level as others were viewed as unproblematic and customary behaviours. Drinking on your own, drinking to escape from reality and drinking excessively were seen as a more problematic drinking behaviour that was not as socially accepted.

The women voiced that there were gender and age differences in anticipated drinking behaviour. Among youths, the expectations were generally to become drunk, whereas adult women were expected to keep control and drink a few glasses. The older study participants talked about outgrowing the intoxication culture prevalent among youths. Although some women found that men drank more frequently or binge drank more often than women, others did not see these differences.

*When you’re in the pub everyone looks equally drunk to me. Almost. (participant 22, group 5)*

*Then it’s so different depending on where you are, what age. I don’t think the same way at all as I did in my 20s. And I didn’t think there was anything wrong with drinking that much and so often back then, so that’s something you… familiarize with and learn from, or not. (participant 21, group 5)*

#### Personal strategies

All the women had experienced situations where they were expected to drink alcohol. This was not always problematic but could be motivating. In these situations, it was common for the women to drink, sometimes unreflected, as this was customary. If the women did not want to drink alcohol, they used different strategies to handle the social expectations. One strategy was to avoid the situation completely by declining parties and nights out. Another strategy was to drink non-alcoholic beer, wine or cocktails without revealing that it was non-alcoholic. Another approach was to provide an excuse, like that they had some health problems, or had to get up early the next morning. For some women, it was easy to state that they did not want to consume alcohol but for others it took a lot of courage.

The women reasoned that if they were to become pregnant and did not want to reveal their pregnancy status, they could use the same strategies as they used before pregnancy. However, in this hypothetic situation, they more often mentioned avoiding drinking situations altogether.

*And sometimes I don’t even say that I haven’t been drinking because no one takes any notice… (participant 33, group 7)*

*I really agree with everything you say because after a few times when you want to wake up fresh the next morning without the day being ruined, “oh, are you pregnant?” is always the first question and when you get fed up with that you just don’t tell anyone anymore and you just mix something, a juice or whatever in a glass and put a cocktail stick in it and everyone thinks it’s a cocktail. (participant 31, group 7)*

### Awareness and uncertainty regarding alcohol and pregnancy

The participants agreed that alcohol consumption during pregnancy can be harmful and were aware that abstinence was recommended. However, they expressed uncertainty regarding the consequences and impact of the amount and timing and wished for more information.

#### Knowledge and misconceptions

All women recognized that alcohol use during pregnancy is harmful and that pregnant women are recommended to abstain from alcohol while pregnant. This knowledge was thought to be generally accepted and most participants could not specify when or where they learnt this. Nevertheless, awareness of specific effects was limited. Only a few women used the term FAS (fetal alcohol sydrome) and were certain about the known consequences of fetal exposure to alcohol.

Although most women figured that heavy alcohol use constitutes a higher risk, a few stated that all exposure independent of amount was equally harmful. A few women had heard that consuming small amounts was harmless but were not confident about the accuracy of these reports. They made it clear that they themselves would not consume alcohol if pregnant or that they would look carefully into the evidence. Much ambivalence was expressed concerning the timing of exposure. In early pregnancy, the embryo is developing fast and should therefore be vulnerable. Even so, based on anecdotes, the participants reasoned that many women have consumed alcohol unknowingly in the early stages of pregnancy and despite that, have had healthy children.

*But it still matters I guess (to drink in early pregnancy) but it gets worse when it is (participant 10, group 3)*

*… a fetus and like I think the injuries are caused more because it lives in the amniotic fluid and all of that more than in the very beginning. (participant 9, group 3)*

*Well I don’t know because it grows so much in the beginning and so on (participant 7, group 3)*

*Yes but it takes up everything you eat … (participant 9, group 3)*

Younger women as well as depressed women were in general thought to be more likely to consume alcohol during pregnancy. Other features that were considered to be risk factors were low self-esteem, low educational level or socioeconomic status, high stress level and having a profession in which alcohol was part of the work culture. Knowledge about alcohol and pregnancy was thought to be so ubiquitous that it was commented recurrently that the study participants could not imagine that anyone would drink alcohol therefore it was hard to discuss risk factors.

#### A need for information

A wish for more information on alcohol consumption during pregnancy was expressed at all interviews. It was assumed that many women who drink around conception or throughout pregnancy are unaware of the associated risks. If provided with reliable information, these women were thought to have the capacity and willingness to reduce their drinking. An intimidation tactic was advocated by some women; others stated the importance of reliable information grounded in research. It was suggested that information on general pregnancy planning as well as the specific impact of alcohol consumption on fertility, pregnancy outcome and child health should be made available all young men and women. Most people who do not plan a pregnancy would not look for such information, therefore it needed to be brought to them. The distribution of this information could be mandatory in elementary schools, through advertising campaigns, via TV programs or health care.

*They focus on the main aspects and not the details but say like ‘if you drink you’ll have a miscarriage’. (participant 12, group 4)*

*Yes it’s good to exaggerate a little bit. (participant 18 group 4)*

*It’s what they do with smoking as well, like showing one of those lungs, but that’s from a lifetime of smoking like not just from the odd smoke… (participant 13, group 4)*

*But you want to know exactly, I feel I want to know the whole truth anyway. But some people might not be able to handle that. (participant 15, group 4)*

### Transition to parenthood

Pregnancy and pregnancy planning were understood as life events that would ease reduction in alcohol consumption. Decreasing alcohol intake was seen as an important part of becoming a parent. The women believed that the partners of pregnant women should also decrease their alcohol consumption, both to support the pregnant woman but also to prepare for the new parental role. Consuming alcohol often or in large quantities was found to be incompatible with being a parent, therefore the transition could influence social life and customs.

#### Responsibility for the child

Most women argued that abstaining from alcohol during pregnancy was the only responsible option. The embryo is exposed to alcohol via the mother and therefore she should abstain in her child’s best interests. The pleasure of consuming alcohol was found to be insignificant compared with the well-being of a child. Not abstaining from alcohol during pregnancy signalled unhealthy drinking habits unsuitable for a prospective parent. This was challenged by a few women who advocated autonomy and voiced that drinking small amounts could be acceptable. One group concluded that pregnant women have the responsibility to consider the scientific evidence before deciding to drink. Occasional associations between alcohol exposure in utero and child abuse were made. Shared responsibility whereby friends, family and societal bodies should intervene to help pregnant women abstain from alcohol was promoted by most women advocating zero tolerance.

The question of when responsibility for the unborn baby starts was complex. On the one hand, many women thought that the responsibility for the child starts with pregnancy planning; if you want to become pregnant then you should avoid alcohol. On the other hand, it was reasoned that it could be difficult not to drink as conceiving could be a long process. When not actively trying to conceive, the women did not see a need to adjust their alcohol consumption. Some women did not think they would change their alcohol consumption before they actually knew that they were pregnant because they did not want to limit themselves to no avail. Other women figured that drinking in early pregnancy would be most harmful and stated that they would reduce or stop their alcohol consumption if trying to become pregnant.

*But I don’t think you can say that it’s your own responsibility. Or it is your own responsibility but it’s not just your own body but there really is somebody else there as well. It’s so much easier to talk about taking responsibility when you talk about, well, normal alcohol habits and it gets so much more complicated when it’s this little innocent thing (the fetus). (participant 25, group 6)*

#### Consequences for social life

Planning to become pregnant was seen as a new stage in life. The assumed impact of this change on social life varied with the current social situation of the women. For women who drank often in order to become drunk, pregnancy would lead to major changes in their social life. For women who typically drank small amounts, the effects of becoming pregnant did not seem as striking. Moreover, the participants expressed that women who always drank in social situations when not pregnant were more likely to avoid going to a party if they became pregnant.

Although some women figured that having a baby would have a drastic impact on life for both mothers and fathers, others argued that it was possible to combine being a parent with current activities. Pregnancy was found to be a good period to prepare for parenthood. Generally, it was found acceptable that the partner drank small to moderate amounts during pregnancy but it should be in a responsible manner because prospective parents should prioritize the family.

*I think maybe that you can get left out when you’re young and pregnant and your friends mostly are partying at the weekends and you can get a bit lonely. (participant 3, group 1)*

*Some maybe wait to have a baby because they have friends who party and so on. (participant 1, group 1)*

## Discussion

The aim of this study was to explore the voice of non-pregnant women concerning alcohol consumption in relation to pregnancy. The participants experienced that it was socially expected that women of fertile age consume alcohol. Simultaneously, during pregnancy there was a contrasting expectation for total abstinence. The participants did not have children and were not pregnant, and their knowledge on the effects of alcohol use during pregnancy was sparse. However, zero tolerance on alcohol was the by far most advocated strategy for a pregnant woman.

In the theme, an issue that cannot be ignored, most of the discussions on alcohol consumption initiated by the women reflected enjoyment. Similar findings have been reported in Denmark where both men and women perceived alcohol as positive and attractive for social reasons [21]. In the present study, drinking according to the expectations prevalent for a certain group or situation was found to be unproblematic. For some women, binge drinking was unproblematic but drinking to relax was a concern and vice versa. These differing views on consumption could be important from a preventive perspective. Women who drink frequently, but not necessarily large amounts, are likely to have formed a stronger habit, potentially more difficult to break during pregnancy [9] therefore they might have an increased risk for continued moderate consumption during pregnancy. Conversely, women who drink less frequently but in a binge drinking pattern have an increased risk of exposing their fetus to high peak doses of alcohol before pregnancy recognition. Preventive strategies directed at individuals with increased risk, i.e. preventive measures [22] are useful within antenatal care for women at high risk of continued consumption during pregnancy. Strategies directed at subgroups with increased risk for binge drinking in early pregnancy could be appropriate in other settings such as youth clinics, primary care or contraceptive counselling.

According to our results, the participants were confident in their ability to stop drinking if they wanted to or if they were to become pregnant. Intention to perform a behaviour and belief in one’s own capability to perform the behaviour are important factors for behaviour change according to sociocognitive theories such as Social Cognitive Theory [23] and Theory of Planned Behaviour [24]. The intention is influenced by thoughts about other’s opinion regarding the behaviour. For example, social expectations on abstinence during pregnancy and belief in one’s ability to cease drinking if becoming pregnant could influence actual behaviour when becoming pregnant. Social cognitive theories do not consider the impact of automatic responses to contextual cues, i.e. habits. However, pregnancy can be considered a contextual change that can help to break habits [25]. Many study participants agreed with the perceived societal expectation to abstain from alcohol during pregnancy. The conflicting expectations on women’s use of alcohol depending on pregnancy status were thought to create most problems for women not wanting to disclose that they are pregnant. This is in line with an Australian study in which pregnant women reported that the conflict between the drinking norm and the good mother norm made them reveal their pregnancy status to avoid being pressured to drink and/or using strategic ways to hide that they were not drinking [11]. One possible way for women to reduce this role conflict is to decrease their alcohol consumption and occasionally break the drinking norm before deciding to conceive.

The abstinence message given to pregnant women via antenatal care was supported by many participants. However, as reflected in the theme, awareness and uncertainty regarding alcohol and pregnancy, most were uncertain of the risks of alcohol consumption around conception and in early pregnancy. They were unsure about specific outcomes related to alcohol exposure during pregnancy and only a few used the term FAS. This can be compared with an Australian focus group study with pregnant women, their partners and new mothers who had varying degrees of knowledge on FAS [26]. In contrast, participants in an American focus group study with pregnant and non-pregnant women could describe the problems caused by alcohol exposure during pregnancy in all 20 of the groups included in the study [10]. The women in the present study who expressed knowledge about FAS and specific outcomes had all gained their knowledge at university, which indicates that this information is not widely distributed among young women.

The participants requested more information on alcohol and pregnancy delivered as a matter of public health. Scare tactics were advocated by a few women; others encouraged honesty and accurate information. The conflict between recommending abstinence based on the precautionary principle and delivering information on the lack of evidence on the effects of low alcohol intake during pregnancy has been discussed by health care ethicist, Gavaghan [27], who argues that women may lose trust in recommendations if they find the danger over exaggerated.

Younger age was mentioned as a risk factor for drinking during pregnancy. These thoughts are not in line with empirical findings from several countries where older women have been found to drink to a greater extent [28]. Furthermore, studies on socioeconomic status show that it is more common for women with higher income or higher social class [28] to drink during pregnancy, whereas our participants figured that lower socioeconomic status could mean a higher risk.

The findings in the theme, transition to parenthood, are in contrast to findings from studies of pregnant women [13, 26, 29]; the non-pregnant participants in our study did not believe that any amount of alcohol use during pregnancy was harmless. This difference might be due to cultural differences in the study populations. The study participants found drinking any alcohol was irresponsible and because the fetus could not influence the decision on alcohol, autonomy was considered by many to be irrelevant. It has been argued that prospective non-dependent parents can be considered to take responsibility for their unborn child in the same way as parents are assumed to make decisions for their children [30]. Once again, this discussion highlights the need for the delivery of accurate information in order for prospective parents to make well-informed decisions.

The women expressed that starting a family comes with responsibility for both parents. It has been found to be more important for pregnant women that their partners support her decision to abstain than the partners cut down their own drinking [26]. Life course research has established transition to parenthood as important for changing alcohol behaviour in adulthood [31, 32]. It seems that both men and women decrease alcohol intake during pregnancy and when living with children. Spending less time with people outside the family as well as social norms discouraging alcohol use within a family have been suggested to explain this decrease [31].

### Methodological considerations

By using a focus group design, we have been able to reflect the group interaction in the discussions. Use of stimuli material evoked feelings among the women and the discussions were often vivid.

Using pre-existing groups can be beneficial in focus group research as the members potentially have shared experiences and feel comfortable in expressing opinions. Then again, revealing sensitive information can be harder in a group where participants will meet again [33]. As the subject of alcohol consumption during pregnancy can potentially be sensitive, even for women who have not experienced a pregnancy, the participants were asked to keep the information revealed within the group. However, confidentiality could not be guaranteed, which could have biased the results towards socially accepted responses. The use of convenience and snowball sampling might have skewed the sample towards certain subgroups of non-pregnant women. The women who participated in the interviews may differ from the women who were not interested in participation or who dropped out. It is probable that the participants were more interested in the subject which may have impacted on the results. Furthermore, all discussions were in Swedish, therefore non-Swedish-speaking women were not included. Due to late cancellations, two groups consisted of only three participants. As participant interaction is characteristic for focus group interviews, at least four participants are recommended [15]. However, even in the small groups, it was clear that the participants benefited from the group format as they built on each other’s statements.

The study was conducted with a limited number of non-pregnant women in south east Sweden and the results are not meant to be generalized. However, the results reflect findings in similar studies with pregnant and non-pregnant women from different parts of the world, indicating that some commonalities are present. To enhance the transferability of the results, we have provided information on the context in which the study was performed and described the recruitment, study participants, interview procedure, and analysis procedure as thoroughly as possible [18].

To improve the credibility of the work, participants were recruited from different sites. Furthermore, all the authors involved in the analysis had different competences [15]. The authors and assessors engaged in the study are researchers/ PhD students without any clinical- or school engagements. The first author is a PhD-student with background in public health sciences and experience from studies on alcohol consumption during pregnancy. The second and third authors have previously worked as midwives and have a special interest in parental education and preconception care. Previous experiences and knowledge within the research team may have an impact on formulations of research questions as well as interpretation of the data. To improve the conformability the themes found in the analysis were reviewed in relation to the entire data and illustrative extracts have been provided. Further, a researcher not involved in the study critically examined the themes with regard to the original data.

## Conclusions

Societal expectations on alcohol consumption among women change drastically during pregnancy, from an expectation to drink to an expectation to abstain. The opinions regarding zero tolerance during pregnancy present among the women in this study did not seem to be based on specific knowledge concerning the consequences of fetal alcohol exposure. The intention to drink or abstain during pregnancy might therefore be more influenced by public opinion than by informed risk perception.

## Implications

The findings from the present study indicate that there is a need for public health interventions directed at different populations. As there seem to be a strong societal expectation on women of fertile age to drink alcohol, strategies to help women resist alcohol can potentially reduce alcohol consumption at all stages of life, including while trying to conceive and during pregnancy. This study contributes to an increased understanding of how non-pregnant women of fertile age reason about alcohol in relation to pregnancy. We recommend that information on the risks associated with alcohol consumption at different stages of pregnancy is provided to young men and women.
